# *catena*-Poly[[{diaqua­[2-(4-hy­droxy­phen­yl)acetato-κ^2^ *O*,*O*′]cerium(III)}-bis­[μ-2-(4-hy­droxy­phen­yl)acetato]-κ^3^ *O*,*O*′:*O*;κ^3^ *O*:*O*,*O*′] 4,4′-bipyridine disolvate monohydrate]

**DOI:** 10.1107/S1600536810048166

**Published:** 2010-11-27

**Authors:** Jia-Lu Liu, Jian-Feng Liu, Guo-Liang Zhao

**Affiliations:** a College of Chemistry and Life Sciences, Zhejiang Normal University, Jinhua 321004, People’s Republic of China, and, Zhejiang Normal University Xingzhi College, Jinhua 321004, People’s Republic of China

## Abstract

In the polymeric title complex, {[Ce(C_8_H_7_O_3_)_3_(H_2_O)_2_]·2C_10_H_8_N_2_·H_2_O}_*n*_, the Ce^III^ ion is coordinated by ten O atoms from four 2-(4-hy­droxy­phen­yl)acetate (HPAA) ligands and two water mol­ecules. One HPAA ligand coordinates just to one Ce^III^ ion, whereas the remaining two bridge two Ce^III^ ions. The 4,4′-bipyridine mol­ecule and one water mol­ecule are not coordinated to Ce. In the crystal, O—H⋯O and O—H⋯N hydrogen bonds link mol­ecules into a three-dimensional network.

## Related literature

For the applications of carb­oxy­lic mental-organic complexes, see: Wang & Sevov (2008 ▶); Fang & Zhang (2006 ▶); Wang *et al.* (2010 ▶). For a related structure, see: Liu *et al.* (2010 ▶).
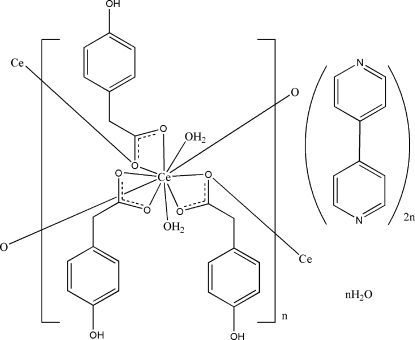


## Experimental

### 

#### Crystal data

[Ce(C_8_H_7_O_3_)_3_(H_2_O)_2_]·2C_10_H_8_N_2_·H_2_O
*M* *_r_* = 959.94
Triclinic, 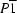

*a* = 9.0793 (2) Å
*b* = 12.8371 (3) Å
*c* = 19.5796 (4) Å
α = 78.534 (1)°
β = 76.716 (1)°
γ = 73.121 (1)°
*V* = 2103.82 (8) Å^3^
*Z* = 2
Mo *K*α radiation
μ = 1.15 mm^−1^
*T* = 296 K
0.32 × 0.16 × 0.06 mm

#### Data collection

Bruker APEXII area-detector diffractometer
Absorption correction: multi-scan (*SADABS*; Sheldrick, 1996 ▶) *T*_min_ = 0.800, *T*_max_ = 0.931
32193 measured reflections
9668 independent reflections
8614 reflections with *I* > 2σ(*I*)
*R*_int_ = 0.034

#### Refinement

*R*[*F* ^2^ > 2σ(*F* ^2^)] = 0.032
*wR*(*F* ^2^) = 0.109
*S* = 0.87
9668 reflections
550 parameters
H-atom parameters constrained
Δρ_max_ = 2.13 e Å^−3^
Δρ_min_ = −0.88 e Å^−3^

### 

Data collection: *APEX2* (Bruker, 2006 ▶); cell refinement: *SAINT* (Bruker, 2006 ▶); data reduction: *SAINT*; program(s) used to solve structure: *SHELXS97* (Sheldrick, 2008 ▶); program(s) used to refine structure: *SHELXL97* (Sheldrick, 2008 ▶); molecular graphics: *XP* in *SHELXTL* (Sheldrick, 2008 ▶); software used to prepare material for publication: *SHELXTL*.

## Supplementary Material

Crystal structure: contains datablocks global, I. DOI: 10.1107/S1600536810048166/bt5395sup1.cif

Structure factors: contains datablocks I. DOI: 10.1107/S1600536810048166/bt5395Isup2.hkl

Additional supplementary materials:  crystallographic information; 3D view; checkCIF report

## Figures and Tables

**Table 1:** Hydrogen-bond geometry (Å, °)

*D*—H⋯*A*	*D*—H	H⋯*A*	*D*⋯*A*	*D*—H⋯*A*
O1*W*—H1⋯O4^i^	0.81	1.96	2.765 (3)	172
O1*W*—H7⋯N1^i^	0.83	2.02	2.774 (4)	151
O2*W*—H2⋯O5^ii^	0.82	1.89	2.703 (3)	177
O2*W*—H5⋯O3*W*^i^	0.82	2.16	2.924 (3)	155
O3—H15*A*⋯N3^iii^	0.86	1.94	2.781 (4)	168
O6—H6*A*⋯O3*W*^iv^	0.82	1.87	2.684 (3)	172
O9—H9*A*⋯N2^v^	0.80	1.91	2.700 (5)	168
O3*W*—H10⋯O2^i^	0.85	1.91	2.752 (3)	168
O3*W*—H11⋯N4^vi^	0.81	2.01	2.817 (4)	175

Symmetry codes: (i) 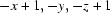; (ii) 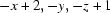; (iii) 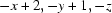; (iv) 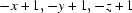; (v) 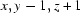; (vi) 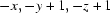.

**Fig. 1.**
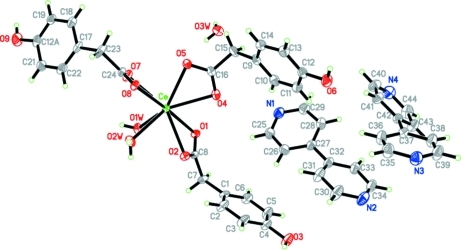
The molecular structure of the title complex, showing the atom-labelling scheme. Displacement ellipsoids are drawn at the 30% probability level.

## supplementary crystallographic information

### Comment

The design and synthesis of carboxylic mental-organic complexes have been an increasing interest for decades owing to their potential practical applications, such as fluorescence, magnetism (Wang, *et al.*, 2010; Fang *et al.*, 2006; Wang, *et al.*, 2008). We have worked at it before (Liu, *et al.*, 2010). In the paper, we report the crystal structure of a new cerium^III^ complex with the ligand *p*- hydroxyphenylacetic acid. The title compound consist of six *L* ligands, two bipy molecules and five water molecules. In the bicentric structure compound, every centric atom is coordinated with nine O atoms from four *L* ligands and two water molecule. The centric atom is nine coordinated. The PAA ligands are coordinated by two modes, bridging and bridging tridentate. (Fig.1). Two 4,4'–bipyridine (bipy) molecules are dissociative.The cerium^III^ atom is in a distort capped pentagonal prism environment. The Ce—O bond length range from 2.5069 (19) Å-2.860 (2) Å. The Ce—O(water) bond length range from 2.5224 (19) Å-2.540 (2) Å, is slightly shorter than other Ce—O bonds. In addition, there are plenty of hydrogen bonds in the crystal structure due to the existence of dissociative water and crystal water molecules. The occurrence of numerous O—H···O involving coordinated and non-coordinated water molecules build up an intricated three dimensionnal network.

### Experimental

All reagents and solvents used were of commercially available quality and without purified before using. *p*-hydroxyphenylacetic acid (HPAA) (0.456 g, 3 mmol) and sodium hydroxide (0.12 g, 3 mmol) were mixed together in water (10 ml), then Ce[(NO_3_)_3_] (0.327 g, 1 mmol) dissolved in water(10 ml) was added into the above solution, after stirring for an hour, an ethanol (5 ml) solution of 4,4'-bipyridine (0.156 g, 1 mmol) was slowly dripped into the above solution with stirring for three hours. After filtration, the filtrate was allowed to stand at room temperature, and single crystals were obtained after one week.

### Refinement

All H atoms   were fixed geometrically and treated as riding with C—H = 0.97 Å (methylene) or 0.93 Å (aromatic) and O—H = 0.82 Å with *U*_iso_(H) = 1.2*U*_eq_(C) or *U*_iso_(H) = 1.5*U*_eq_(O).

### Crystal data

**Table:**

[Ce(C_8_H_7_O_3_)_3_(H_2_O)_2_]·2C_10_H_8_N_2_·H_2_O	*Z* = 2
*M**_r_* = 959.94	*F*(000) = 978
Triclinic, *P*1	*D*_x_ = 1.515 Mg m^−^^3^
Hall symbol: -P 1	Mo *K*α radiation, λ = 0.71073 Å
*a* = 9.0793 (2) Å	Cell parameters from 9983 reflections
*b* = 12.8371 (3) Å	θ = 1.1–27.7°
*c* = 19.5796 (4) Å	µ = 1.15 mm^−^^1^
α = 78.534 (1)°	*T* = 296 K
β = 76.716 (1)°	Block, colourless
γ = 73.121 (1)°	0.32 × 0.16 × 0.06 mm
*V* = 2103.82 (8) Å^3^	

### Data collection

**Table:**

Bruker APEXII area-detector diffractometer	9668 independent reflections
Radiation source: fine-focus sealed tube	8614 reflections with *I* > 2σ(*I*)
graphite	*R*_int_ = 0.034
phi and ω scans	θ_max_ = 27.7°, θ_min_ = 1.1°
Absorption correction: multi-scan (*SADABS*; Sheldrick, 1996)	*h* = −11→11
*T*_min_ = 0.800, *T*_max_ = 0.931	*k* = −16→16
32193 measured reflections	*l* = −25→25

### Refinement

**Table:**

Refinement on *F*^2^	Primary atom site location: structure-invariant direct methods
Least-squares matrix: full	Secondary atom site location: difference Fourier map
*R*[*F*^2^ > 2σ(*F*^2^)] = 0.032	Hydrogen site location: inferred from neighbouring sites
*wR*(*F*^2^) = 0.109	H-atom parameters constrained
*S* = 0.87	*w* = 1/[σ^2^(*F*_o_^2^) + (0.1*P*)^2^] where *P* = (*F*_o_^2^ + 2*F*_c_^2^)/3
9668 reflections	(Δ/σ)_max_ = 0.003
550 parameters	Δρ_max_ = 2.13 e Å^−^^3^
0 restraints	Δρ_min_ = −0.88 e Å^−^^3^

### Special details

**Table:**

Geometry. All e.s.d.'s (except the e.s.d. in the dihedral angle between two l.s. planes) are estimated using the full covariance matrix. The cell e.s.d.'s are taken into account individually in the estimation of e.s.d.'s in distances, angles and torsion angles; correlations between e.s.d.'s in cell parameters are only used when they are defined by crystal symmetry. An approximate (isotropic) treatment of cell e.s.d.'s is used for estimating e.s.d.'s involving l.s. planes.
Refinement. Refinement of *F*^2^ against ALL reflections. The weighted *R*-factor *wR* and goodness of fit *S* are based on *F*^2^, conventional *R*-factors *R* are based on *F*, with *F* set to zero for negative *F*^2^. The threshold expression of *F*^2^ > σ(*F*^2^) is used only for calculating *R*-factors(gt) *etc*. and is not relevant to the choice of reflections for refinement. *R*-factors based on *F*^2^ are statistically about twice as large as those based on *F*, and *R*- factors based on ALL data will be even larger.

### Fractional atomic coordinates and isotropic or equivalent isotropic displacement parameters (Å^2^)

**Table:**

	*x*	*y*	*z*	*U*_iso_*/*U*_eq_	
Ce	0.763886 (13)	−0.024594 (10)	0.502284 (7)	0.02165 (7)	
O1	0.5164 (2)	−0.00623 (18)	0.42935 (11)	0.0311 (4)	
O1W	0.6869 (2)	−0.20312 (17)	0.52006 (11)	0.0342 (5)	
H1	0.5949	−0.2024	0.5266	0.051*	
H7	0.7230	−0.2533	0.5500	0.051*	
O2	0.7605 (2)	−0.04863 (18)	0.37493 (11)	0.0330 (4)	
O2W	0.9859 (2)	−0.19000 (18)	0.46923 (13)	0.0405 (5)	
H2	1.0714	−0.1762	0.4589	0.061*	
H5	0.9845	−0.2218	0.4370	0.061*	
O3	0.7135 (3)	0.1785 (3)	0.04269 (14)	0.0669 (8)	
H15A	0.8121	0.1711	0.0295	0.100*	
O4	0.6313 (2)	0.17990 (17)	0.46325 (12)	0.0353 (5)	
O5	0.7312 (2)	0.14537 (17)	0.55952 (11)	0.0331 (4)	
O3W	0.0235 (3)	0.2327 (2)	0.67074 (13)	0.0496 (6)	
H10	0.0906	0.1720	0.6632	0.074*	
H11	−0.0512	0.2250	0.7009	0.074*	
O6	0.8176 (3)	0.6417 (2)	0.29794 (13)	0.0520 (6)	
H6A	0.8648	0.6770	0.3113	0.078*	
O7	0.8060 (2)	−0.1038 (2)	0.62835 (11)	0.0379 (5)	
O8	1.0105 (2)	−0.04972 (17)	0.56681 (11)	0.0300 (4)	
O9	0.8706 (4)	−0.4737 (2)	0.88856 (16)	0.0643 (8)	
H9A	0.8225	−0.4589	0.9268	0.096*	
N1	0.3011 (4)	0.3471 (3)	0.35414 (16)	0.0527 (8)	
N2	0.7018 (5)	0.5461 (3)	0.02036 (19)	0.0644 (9)	
N3	0.9775 (4)	0.8207 (3)	0.01648 (18)	0.0590 (8)	
N4	0.2342 (4)	0.8067 (3)	0.2258 (2)	0.0623 (9)	
C1	0.6107 (3)	0.0024 (3)	0.24341 (15)	0.0351 (6)	
C2	0.7638 (4)	0.0005 (4)	0.21124 (18)	0.0534 (10)	
H2A	0.8446	−0.0417	0.2346	0.064*	
C3	0.8000 (4)	0.0595 (4)	0.14557 (18)	0.0558 (10)	
H3A	0.9041	0.0574	0.1258	0.067*	
C4	0.6838 (4)	0.1215 (3)	0.10881 (17)	0.0471 (8)	
C5	0.5313 (4)	0.1251 (4)	0.1398 (2)	0.0576 (10)	
H5A	0.4509	0.1669	0.1161	0.069*	
C6	0.4958 (4)	0.0662 (3)	0.20698 (19)	0.0493 (9)	
H6C	0.3915	0.0705	0.2275	0.059*	
C7	0.5671 (4)	−0.0639 (3)	0.31479 (16)	0.0380 (7)	
H7A	0.4544	−0.0530	0.3253	0.046*	
H7C	0.6139	−0.1413	0.3111	0.046*	
C8	0.6167 (3)	−0.0365 (2)	0.37653 (14)	0.0266 (5)	
C9	0.6708 (3)	0.4103 (2)	0.46172 (18)	0.0374 (7)	
C10	0.6231 (4)	0.4333 (3)	0.3971 (2)	0.0457 (8)	
H10A	0.5557	0.3960	0.3895	0.055*	
C11	0.6725 (4)	0.5099 (3)	0.3433 (2)	0.0451 (8)	
H11A	0.6381	0.5238	0.3004	0.054*	
C12	0.7733 (4)	0.5663 (3)	0.35321 (18)	0.0388 (7)	
C12A	0.9018 (4)	−0.3842 (3)	0.84258 (19)	0.0434 (8)	
C13	0.8237 (4)	0.5439 (3)	0.41697 (19)	0.0431 (7)	
H13A	0.8920	0.5808	0.4242	0.052*	
C14	0.7731 (4)	0.4667 (3)	0.4704 (2)	0.0445 (7)	
H14A	0.8084	0.4523	0.5132	0.053*	
C15	0.6064 (4)	0.3334 (3)	0.5228 (2)	0.0488 (8)	
H15B	0.6356	0.3420	0.5656	0.059*	
H15C	0.4930	0.3556	0.5295	0.059*	
C16	0.6609 (3)	0.2132 (2)	0.51396 (16)	0.0296 (6)	
C17	0.9711 (4)	−0.2083 (3)	0.74364 (16)	0.0393 (7)	
C18	0.9054 (4)	−0.1947 (3)	0.81334 (18)	0.0437 (8)	
H18B	0.8836	−0.1259	0.8273	0.052*	
C19	0.8712 (4)	−0.2823 (3)	0.86307 (17)	0.0431 (8)	
H19A	0.8279	−0.2720	0.9099	0.052*	
C21	0.9695 (5)	−0.3997 (3)	0.7736 (2)	0.0504 (9)	
H21A	0.9923	−0.4688	0.7598	0.061*	
C22	1.0034 (4)	−0.3116 (3)	0.72483 (19)	0.0482 (9)	
H22A	1.0490	−0.3225	0.6783	0.058*	
C23	1.0119 (5)	−0.1149 (4)	0.68953 (18)	0.0559 (11)	
H23A	0.9810	−0.0493	0.7123	0.067*	
H23B	1.1247	−0.1321	0.6744	0.067*	
C24	0.9378 (3)	−0.0893 (2)	0.62473 (14)	0.0281 (6)	
C25	0.4182 (6)	0.2834 (3)	0.3156 (2)	0.0647 (11)	
H25A	0.4475	0.2090	0.3332	0.078*	
C26	0.5000 (6)	0.3192 (4)	0.2510 (2)	0.0621 (12)	
H26A	0.5841	0.2704	0.2274	0.075*	
C27	0.4563 (4)	0.4281 (3)	0.22159 (19)	0.0436 (8)	
C28	0.3305 (4)	0.4953 (3)	0.2606 (2)	0.0531 (9)	
H28A	0.2951	0.5693	0.2433	0.064*	
C29	0.2579 (5)	0.4513 (4)	0.3255 (2)	0.0559 (10)	
H29A	0.1735	0.4979	0.3507	0.067*	
C30	0.7752 (6)	0.4681 (5)	0.0663 (2)	0.0732 (14)	
H30A	0.8823	0.4385	0.0532	0.088*	
C31	0.7007 (5)	0.4287 (4)	0.1324 (2)	0.0635 (12)	
H31A	0.7574	0.3749	0.1627	0.076*	
C32	0.5425 (5)	0.4699 (3)	0.15248 (19)	0.0460 (8)	
C33	0.4652 (5)	0.5511 (3)	0.1053 (2)	0.0529 (9)	
H33A	0.3581	0.5817	0.1172	0.063*	
C34	0.5484 (6)	0.5862 (4)	0.0404 (2)	0.0607 (10)	
H34A	0.4945	0.6404	0.0093	0.073*	
C35	0.9607 (5)	0.7396 (4)	0.0695 (2)	0.0619 (11)	
H35A	1.0503	0.6864	0.0796	0.074*	
C36	0.8189 (4)	0.7299 (3)	0.1101 (2)	0.0562 (10)	
H36A	0.8143	0.6717	0.1467	0.067*	
C37	0.6828 (4)	0.8073 (3)	0.09630 (18)	0.0427 (7)	
C38	0.7003 (5)	0.8910 (3)	0.0407 (2)	0.0563 (10)	
H38A	0.6129	0.9446	0.0285	0.068*	
C39	0.8473 (5)	0.8943 (4)	0.0038 (2)	0.0643 (11)	
H39A	0.8559	0.9522	−0.0327	0.077*	
C40	0.3649 (5)	0.7732 (4)	0.2527 (2)	0.0581 (10)	
H40A	0.3567	0.7512	0.3014	0.070*	
C41	0.5119 (4)	0.7694 (3)	0.21208 (19)	0.0488 (8)	
H41A	0.6000	0.7429	0.2332	0.059*	
C42	0.5278 (4)	0.8048 (3)	0.14048 (18)	0.0410 (7)	
C43	0.3907 (4)	0.8400 (3)	0.1118 (2)	0.0536 (9)	
H43A	0.3950	0.8637	0.0634	0.064*	
C44	0.2499 (5)	0.8389 (4)	0.1563 (2)	0.0627 (11)	
H44A	0.1599	0.8622	0.1365	0.075*	

### Atomic displacement parameters (Å^2^)

**Table:**

	*U*^11^	*U*^22^	*U*^33^	*U*^12^	*U*^13^	*U*^23^
Ce	0.01870 (9)	0.02339 (9)	0.02159 (10)	−0.00760 (6)	−0.00219 (6)	0.00130 (6)
O1	0.0275 (10)	0.0399 (11)	0.0244 (10)	−0.0115 (8)	0.0014 (8)	−0.0041 (9)
O1W	0.0256 (9)	0.0320 (10)	0.0437 (12)	−0.0111 (8)	−0.0075 (8)	0.0049 (9)
O2	0.0260 (10)	0.0457 (12)	0.0287 (11)	−0.0128 (8)	−0.0021 (8)	−0.0066 (9)
O2W	0.0254 (10)	0.0377 (11)	0.0598 (15)	−0.0059 (8)	−0.0101 (9)	−0.0110 (10)
O3	0.0549 (16)	0.095 (2)	0.0399 (15)	−0.0192 (15)	−0.0099 (12)	0.0170 (15)
O4	0.0280 (10)	0.0320 (10)	0.0452 (13)	−0.0091 (8)	−0.0090 (9)	0.0011 (9)
O5	0.0275 (10)	0.0313 (10)	0.0397 (12)	−0.0059 (8)	−0.0066 (8)	−0.0052 (9)
O3W	0.0434 (13)	0.0503 (14)	0.0555 (16)	−0.0184 (11)	0.0023 (11)	−0.0118 (12)
O6	0.0633 (16)	0.0468 (14)	0.0495 (15)	−0.0268 (12)	−0.0112 (12)	0.0051 (12)
O7	0.0308 (11)	0.0530 (13)	0.0316 (11)	−0.0219 (10)	−0.0087 (8)	0.0102 (10)
O8	0.0277 (10)	0.0363 (11)	0.0238 (10)	−0.0131 (8)	−0.0024 (8)	0.0050 (8)
O9	0.079 (2)	0.0490 (15)	0.0510 (17)	−0.0221 (14)	0.0018 (14)	0.0174 (13)
N1	0.0580 (19)	0.0592 (19)	0.0413 (17)	−0.0253 (16)	−0.0049 (14)	0.0023 (15)
N2	0.074 (2)	0.078 (2)	0.0431 (18)	−0.039 (2)	−0.0085 (16)	0.0124 (17)
N3	0.0514 (18)	0.063 (2)	0.051 (2)	−0.0127 (16)	0.0030 (14)	−0.0007 (16)
N4	0.053 (2)	0.068 (2)	0.064 (2)	−0.0255 (17)	0.0012 (16)	−0.0064 (18)
C1	0.0346 (15)	0.0517 (18)	0.0245 (15)	−0.0169 (13)	−0.0033 (11)	−0.0116 (13)
C2	0.0315 (16)	0.095 (3)	0.0296 (17)	−0.0184 (18)	−0.0070 (13)	0.0055 (18)
C3	0.0309 (16)	0.098 (3)	0.0324 (18)	−0.0164 (18)	−0.0039 (13)	0.0019 (19)
C4	0.0459 (19)	0.065 (2)	0.0280 (17)	−0.0172 (16)	−0.0064 (13)	0.0021 (16)
C5	0.0355 (18)	0.079 (3)	0.047 (2)	−0.0073 (17)	−0.0085 (15)	0.007 (2)
C6	0.0293 (16)	0.071 (2)	0.042 (2)	−0.0117 (15)	−0.0023 (13)	−0.0030 (17)
C7	0.0358 (16)	0.0577 (19)	0.0272 (15)	−0.0239 (14)	−0.0020 (12)	−0.0079 (14)
C8	0.0269 (13)	0.0311 (13)	0.0226 (13)	−0.0129 (10)	−0.0026 (10)	0.0002 (11)
C9	0.0349 (15)	0.0252 (13)	0.0465 (19)	−0.0042 (11)	−0.0014 (13)	−0.0044 (13)
C10	0.0454 (18)	0.0369 (16)	0.059 (2)	−0.0154 (14)	−0.0110 (16)	−0.0078 (16)
C11	0.051 (2)	0.0435 (18)	0.047 (2)	−0.0169 (15)	−0.0166 (16)	−0.0032 (15)
C12	0.0371 (16)	0.0329 (15)	0.0445 (18)	−0.0104 (12)	−0.0033 (13)	−0.0035 (13)
C12A	0.0363 (17)	0.0494 (19)	0.0364 (18)	−0.0122 (14)	−0.0058 (13)	0.0134 (15)
C13	0.0400 (17)	0.0414 (17)	0.053 (2)	−0.0170 (14)	−0.0103 (14)	−0.0056 (15)
C14	0.0490 (19)	0.0403 (17)	0.044 (2)	−0.0108 (14)	−0.0108 (15)	−0.0045 (14)
C15	0.056 (2)	0.0272 (14)	0.052 (2)	−0.0065 (14)	0.0071 (16)	−0.0065 (14)
C16	0.0182 (12)	0.0291 (13)	0.0388 (16)	−0.0082 (10)	0.0019 (11)	−0.0037 (12)
C17	0.0375 (16)	0.0552 (19)	0.0288 (16)	−0.0237 (14)	−0.0156 (12)	0.0146 (14)
C18	0.0474 (18)	0.0488 (18)	0.0356 (18)	−0.0180 (15)	−0.0133 (14)	0.0073 (15)
C19	0.0453 (18)	0.0497 (19)	0.0266 (16)	−0.0105 (15)	−0.0030 (13)	0.0057 (14)
C21	0.058 (2)	0.0448 (19)	0.040 (2)	−0.0069 (17)	−0.0055 (16)	0.0014 (16)
C22	0.046 (2)	0.063 (2)	0.0276 (17)	−0.0145 (17)	−0.0009 (14)	0.0043 (16)
C23	0.067 (2)	0.082 (3)	0.0358 (19)	−0.055 (2)	−0.0277 (17)	0.0274 (18)
C24	0.0292 (13)	0.0298 (13)	0.0255 (14)	−0.0114 (11)	−0.0071 (10)	0.0038 (11)
C25	0.092 (3)	0.044 (2)	0.047 (2)	−0.019 (2)	0.003 (2)	0.0051 (18)
C26	0.088 (3)	0.042 (2)	0.045 (2)	−0.016 (2)	0.006 (2)	−0.0031 (17)
C27	0.053 (2)	0.0472 (19)	0.0366 (18)	−0.0232 (16)	−0.0125 (15)	0.0018 (15)
C28	0.057 (2)	0.047 (2)	0.053 (2)	−0.0160 (17)	−0.0134 (17)	0.0054 (17)
C29	0.046 (2)	0.066 (2)	0.049 (2)	−0.0094 (18)	−0.0044 (17)	−0.0048 (19)
C30	0.060 (3)	0.105 (4)	0.048 (2)	−0.029 (3)	−0.0100 (19)	0.016 (2)
C31	0.054 (2)	0.084 (3)	0.045 (2)	−0.021 (2)	−0.0115 (18)	0.016 (2)
C32	0.058 (2)	0.0497 (19)	0.0349 (18)	−0.0256 (17)	−0.0093 (15)	0.0030 (15)
C33	0.061 (2)	0.051 (2)	0.046 (2)	−0.0181 (17)	−0.0127 (17)	0.0042 (17)
C34	0.085 (3)	0.055 (2)	0.045 (2)	−0.026 (2)	−0.020 (2)	0.0098 (18)
C35	0.050 (2)	0.060 (2)	0.064 (3)	−0.0048 (18)	−0.0095 (19)	0.004 (2)
C36	0.052 (2)	0.053 (2)	0.055 (2)	−0.0130 (17)	−0.0110 (17)	0.0115 (18)
C37	0.0490 (19)	0.0445 (18)	0.0346 (17)	−0.0112 (14)	−0.0092 (14)	−0.0052 (14)
C38	0.050 (2)	0.056 (2)	0.049 (2)	−0.0050 (17)	−0.0049 (16)	0.0071 (18)
C39	0.059 (2)	0.067 (3)	0.050 (2)	−0.012 (2)	0.0002 (18)	0.013 (2)
C40	0.062 (2)	0.074 (3)	0.043 (2)	−0.032 (2)	−0.0031 (17)	−0.0033 (19)
C41	0.054 (2)	0.059 (2)	0.0400 (19)	−0.0263 (17)	−0.0119 (15)	−0.0023 (17)
C42	0.0478 (18)	0.0409 (17)	0.0368 (18)	−0.0143 (14)	−0.0087 (14)	−0.0053 (14)
C43	0.051 (2)	0.062 (2)	0.045 (2)	−0.0121 (18)	−0.0144 (17)	0.0013 (18)
C44	0.046 (2)	0.068 (3)	0.072 (3)	−0.0138 (19)	−0.0143 (19)	−0.002 (2)

### Geometric parameters (Å, °)

**Table:**

Ce—O8^i^	2.5069 (19)	C11—C12	1.385 (4)
Ce—O1W	2.5224 (19)	C11—H11A	0.9300
Ce—O2W	2.540 (2)	C12—C13	1.377 (5)
Ce—O7	2.546 (2)	C12A—C19	1.377 (5)
Ce—O1^ii^	2.546 (2)	C12A—C21	1.377 (5)
Ce—O5	2.559 (2)	C13—C14	1.386 (5)
Ce—O2	2.581 (2)	C13—H13A	0.9300
Ce—O4	2.591 (2)	C14—H14A	0.9300
Ce—O8	2.726 (2)	C15—C16	1.510 (4)
Ce—O1	2.860 (2)	C15—H15B	0.9700
Ce—C16	2.961 (3)	C15—H15C	0.9700
O1—C8	1.254 (3)	C17—C22	1.378 (6)
O1—Ce^ii^	2.546 (2)	C17—C18	1.381 (5)
O1W—H1	0.8129	C17—C23	1.506 (4)
O1W—H7	0.8259	C18—C19	1.391 (4)
O2—C8	1.263 (3)	C18—H18B	0.9300
O2W—H2	0.8167	C19—H19A	0.9300
O2W—H5	0.8211	C21—C22	1.387 (5)
O3—C4	1.364 (4)	C21—H21A	0.9300
O3—H15A	0.8554	C22—H22A	0.9300
O4—C16	1.265 (4)	C23—C24	1.506 (4)
O5—C16	1.261 (3)	C23—H23A	0.9700
O3W—H10	0.8542	C23—H23B	0.9700
O3W—H11	0.8061	C25—C26	1.377 (6)
O6—C12	1.371 (4)	C25—H25A	0.9300
O6—H6A	0.8173	C26—C27	1.381 (5)
O7—C24	1.248 (3)	C26—H26A	0.9300
O8—C24	1.266 (3)	C27—C28	1.386 (5)
O8—Ce^i^	2.5069 (19)	C27—C32	1.483 (5)
O9—C12A	1.368 (4)	C28—C29	1.384 (5)
O9—H9A	0.8033	C28—H28A	0.9300
N1—C25	1.320 (5)	C29—H29A	0.9300
N1—C29	1.325 (6)	C30—C31	1.388 (6)
N2—C34	1.331 (6)	C30—H30A	0.9300
N2—C30	1.336 (6)	C31—C32	1.371 (6)
N3—C39	1.320 (5)	C31—H31A	0.9300
N3—C35	1.332 (5)	C32—C33	1.386 (5)
N4—C44	1.328 (6)	C33—C34	1.385 (5)
N4—C40	1.331 (5)	C33—H33A	0.9300
C1—C6	1.372 (5)	C34—H34A	0.9300
C1—C2	1.384 (4)	C35—C36	1.374 (6)
C1—C7	1.517 (4)	C35—H35A	0.9300
C2—C3	1.378 (5)	C36—C37	1.386 (5)
C2—H2A	0.9300	C36—H36A	0.9300
C3—C4	1.378 (5)	C37—C38	1.385 (5)
C3—H3A	0.9300	C37—C42	1.479 (5)
C4—C5	1.370 (5)	C38—C39	1.373 (6)
C5—C6	1.399 (5)	C38—H38A	0.9300
C5—H5A	0.9300	C39—H39A	0.9300
C6—H6C	0.9300	C40—C41	1.381 (5)
C7—C8	1.517 (4)	C40—H40A	0.9300
C7—H7A	0.9700	C41—C42	1.373 (5)
C7—H7C	0.9700	C41—H41A	0.9300
C9—C10	1.381 (5)	C42—C43	1.401 (5)
C9—C14	1.388 (4)	C43—C44	1.374 (6)
C9—C15	1.514 (4)	C43—H43A	0.9300
C10—C11	1.380 (5)	C44—H44A	0.9300
C10—H10A	0.9300		
			
O8^i^—Ce—O1W	136.43 (7)	C10—C11—C12	120.1 (3)
O8^i^—Ce—O2W	73.65 (7)	C10—C11—H11A	120.0
O1W—Ce—O2W	64.74 (7)	C12—C11—H11A	120.0
O8^i^—Ce—O7	110.55 (6)	O6—C12—C13	123.6 (3)
O1W—Ce—O7	78.19 (7)	O6—C12—C11	117.4 (3)
O2W—Ce—O7	83.99 (8)	C13—C12—C11	119.0 (3)
O8^i^—Ce—O1^ii^	150.34 (6)	O9—C12A—C19	122.8 (3)
O1W—Ce—O1^ii^	72.33 (7)	O9—C12A—C21	117.2 (4)
O2W—Ce—O1^ii^	136.01 (7)	C19—C12A—C21	120.0 (3)
O7—Ce—O1^ii^	78.23 (7)	C12—C13—C14	120.2 (3)
O8^i^—Ce—O5	77.23 (7)	C12—C13—H13A	119.9
O1W—Ce—O5	143.84 (7)	C14—C13—H13A	119.9
O2W—Ce—O5	136.14 (6)	C13—C14—C9	121.6 (3)
O7—Ce—O5	76.35 (7)	C13—C14—H14A	119.2
O1^ii^—Ce—O5	77.63 (7)	C9—C14—H14A	119.2
O8^i^—Ce—O2	78.68 (6)	C16—C15—C9	115.2 (3)
O1W—Ce—O2	77.07 (7)	C16—C15—H15B	108.5
O2W—Ce—O2	72.36 (7)	C9—C15—H15B	108.5
O7—Ce—O2	151.26 (8)	C16—C15—H15C	108.5
O1^ii^—Ce—O2	107.60 (6)	C9—C15—H15C	108.5
O5—Ce—O2	132.24 (7)	H15B—C15—H15C	107.5
O8^i^—Ce—O4	76.99 (6)	O5—C16—O4	120.1 (3)
O1W—Ce—O4	134.93 (6)	O5—C16—C15	119.1 (3)
O2W—Ce—O4	145.40 (7)	O4—C16—C15	120.7 (3)
O7—Ce—O4	123.87 (8)	O5—C16—Ce	59.33 (14)
O1^ii^—Ce—O4	74.92 (7)	O4—C16—Ce	60.83 (14)
O5—Ce—O4	50.30 (6)	C15—C16—Ce	177.9 (2)
O2—Ce—O4	84.35 (7)	C22—C17—C18	118.0 (3)
O8^i^—Ce—O8	61.86 (7)	C22—C17—C23	120.2 (3)
O1W—Ce—O8	110.71 (6)	C18—C17—C23	121.8 (4)
O2W—Ce—O8	68.19 (7)	C17—C18—C19	121.2 (3)
O7—Ce—O8	48.80 (6)	C17—C18—H18B	119.4
O1^ii^—Ce—O8	121.96 (7)	C19—C18—H18B	119.4
O5—Ce—O8	69.35 (6)	C12A—C19—C18	119.7 (3)
O2—Ce—O8	130.05 (6)	C12A—C19—H19A	120.2
O4—Ce—O8	112.67 (6)	C18—C19—H19A	120.2
O8^i^—Ce—O1	116.84 (6)	C12A—C21—C22	119.5 (4)
O1W—Ce—O1	68.04 (6)	C12A—C21—H21A	120.2
O2W—Ce—O1	108.78 (6)	C22—C21—H21A	120.2
O7—Ce—O1	132.61 (6)	C17—C22—C21	121.6 (4)
O1^ii^—Ce—O1	60.72 (7)	C17—C22—H22A	119.2
O5—Ce—O1	113.44 (6)	C21—C22—H22A	119.2
O2—Ce—O1	47.09 (6)	C24—C23—C17	115.4 (3)
O4—Ce—O1	69.22 (6)	C24—C23—H23A	108.4
O8—Ce—O1	176.85 (5)	C17—C23—H23A	108.4
O8^i^—Ce—C16	75.45 (7)	C24—C23—H23B	108.4
O1W—Ce—C16	147.01 (7)	C17—C23—H23B	108.4
O2W—Ce—C16	148.25 (7)	H23A—C23—H23B	107.5
O7—Ce—C16	100.25 (8)	O7—C24—O8	120.7 (2)
O1^ii^—Ce—C16	75.09 (7)	O7—C24—C23	120.9 (3)
O5—Ce—C16	25.09 (7)	O8—C24—C23	118.4 (2)
O2—Ce—C16	108.46 (8)	N1—C25—C26	124.6 (4)
O4—Ce—C16	25.22 (7)	N1—C25—H25A	117.7
O8—Ce—C16	90.84 (7)	C26—C25—H25A	117.7
O1—Ce—C16	91.55 (7)	C25—C26—C27	119.6 (4)
C8—O1—Ce^ii^	150.02 (17)	C25—C26—H26A	120.2
C8—O1—Ce	89.20 (15)	C27—C26—H26A	120.2
Ce^ii^—O1—Ce	119.28 (7)	C26—C27—C28	116.4 (3)
Ce—O1W—H1	119.7	C26—C27—C32	121.1 (4)
Ce—O1W—H7	118.2	C28—C27—C32	122.5 (3)
H1—O1W—H7	104.3	C29—C28—C27	119.4 (4)
C8—O2—Ce	102.36 (17)	C29—C28—H28A	120.3
Ce—O2W—H2	112.6	C27—C28—H28A	120.3
Ce—O2W—H5	120.1	N1—C29—C28	124.2 (4)
H2—O2W—H5	104.2	N1—C29—H29A	117.9
C4—O3—H15A	109.7	C28—C29—H29A	117.9
C16—O4—Ce	93.95 (16)	N2—C30—C31	123.7 (5)
C16—O5—Ce	95.58 (17)	N2—C30—H30A	118.1
H10—O3W—H11	113.1	C31—C30—H30A	118.1
C12—O6—H6A	109.4	C32—C31—C30	119.2 (4)
C24—O7—Ce	99.76 (16)	C32—C31—H31A	120.4
C24—O8—Ce^i^	150.65 (18)	C30—C31—H31A	120.4
C24—O8—Ce	90.69 (15)	C31—C32—C33	117.6 (4)
Ce^i^—O8—Ce	118.14 (7)	C31—C32—C27	121.4 (3)
C12A—O9—H9A	113.2	C33—C32—C27	121.1 (4)
C25—N1—C29	115.8 (3)	C34—C33—C32	119.6 (4)
C34—N2—C30	116.7 (4)	C34—C33—H33A	120.2
C39—N3—C35	116.1 (3)	C32—C33—H33A	120.2
C44—N4—C40	116.7 (3)	N2—C34—C33	123.2 (4)
C6—C1—C2	116.9 (3)	N2—C34—H34A	118.4
C6—C1—C7	120.0 (3)	C33—C34—H34A	118.4
C2—C1—C7	123.1 (3)	N3—C35—C36	123.8 (4)
C3—C2—C1	121.8 (3)	N3—C35—H35A	118.1
C3—C2—H2A	119.1	C36—C35—H35A	118.1
C1—C2—H2A	119.1	C35—C36—C37	119.7 (4)
C2—C3—C4	120.8 (3)	C35—C36—H36A	120.1
C2—C3—H3A	119.6	C37—C36—H36A	120.1
C4—C3—H3A	119.6	C38—C37—C36	116.3 (3)
O3—C4—C5	118.4 (3)	C38—C37—C42	121.3 (3)
O3—C4—C3	123.1 (3)	C36—C37—C42	122.3 (3)
C5—C4—C3	118.5 (3)	C39—C38—C37	119.6 (4)
C4—C5—C6	120.2 (3)	C39—C38—H38A	120.2
C4—C5—H5A	119.9	C37—C38—H38A	120.2
C6—C5—H5A	119.9	N3—C39—C38	124.4 (4)
C1—C6—C5	121.8 (3)	N3—C39—H39A	117.8
C1—C6—H6C	119.1	C38—C39—H39A	117.8
C5—C6—H6C	119.1	N4—C40—C41	123.3 (4)
C8—C7—C1	115.4 (2)	N4—C40—H40A	118.3
C8—C7—H7A	108.4	C41—C40—H40A	118.3
C1—C7—H7A	108.4	C42—C41—C40	119.9 (3)
C8—C7—H7C	108.4	C42—C41—H41A	120.1
C1—C7—H7C	108.4	C40—C41—H41A	120.1
H7A—C7—H7C	107.5	C41—C42—C43	117.1 (3)
O1—C8—O2	120.9 (2)	C41—C42—C37	121.1 (3)
O1—C8—C7	120.1 (2)	C43—C42—C37	121.9 (3)
O2—C8—C7	119.0 (2)	C44—C43—C42	118.8 (4)
C10—C9—C14	117.1 (3)	C44—C43—H43A	120.6
C10—C9—C15	122.0 (3)	C42—C43—H43A	120.6
C14—C9—C15	120.7 (3)	N4—C44—C43	124.2 (4)
C11—C10—C9	122.0 (3)	N4—C44—H44A	117.9
C11—C10—H10A	119.0	C43—C44—H44A	117.9
C9—C10—H10A	119.0		
			
O8^i^—Ce—O1—C8	43.46 (19)	C15—C9—C10—C11	174.8 (3)
O1W—Ce—O1—C8	−88.73 (17)	C9—C10—C11—C12	0.2 (6)
O2W—Ce—O1—C8	−37.27 (17)	C10—C11—C12—O6	−179.7 (3)
O7—Ce—O1—C8	−136.62 (16)	C10—C11—C12—C13	0.5 (5)
O1^ii^—Ce—O1—C8	−170.2 (2)	O6—C12—C13—C14	179.8 (3)
O5—Ce—O1—C8	130.56 (16)	C11—C12—C13—C14	−0.5 (5)
O2—Ce—O1—C8	3.87 (15)	C12—C13—C14—C9	−0.2 (5)
O4—Ce—O1—C8	105.94 (17)	C10—C9—C14—C13	0.8 (5)
C16—Ce—O1—C8	117.91 (17)	C15—C9—C14—C13	−174.9 (3)
O8^i^—Ce—O1—Ce^ii^	−146.38 (7)	C10—C9—C15—C16	71.5 (4)
O1W—Ce—O1—Ce^ii^	81.43 (9)	C14—C9—C15—C16	−113.1 (4)
O2W—Ce—O1—Ce^ii^	132.89 (9)	Ce—O5—C16—O4	1.2 (3)
O7—Ce—O1—Ce^ii^	33.54 (14)	Ce—O5—C16—C15	178.4 (2)
O1^ii^—Ce—O1—Ce^ii^	0.0	Ce—O4—C16—O5	−1.2 (3)
O5—Ce—O1—Ce^ii^	−59.29 (10)	Ce—O4—C16—C15	−178.3 (2)
O2—Ce—O1—Ce^ii^	174.03 (14)	C9—C15—C16—O5	121.8 (3)
O4—Ce—O1—Ce^ii^	−83.90 (10)	C9—C15—C16—O4	−61.0 (4)
C16—Ce—O1—Ce^ii^	−71.93 (10)	O8^i^—Ce—C16—O5	−90.91 (16)
O8^i^—Ce—O2—C8	−148.50 (18)	O1W—Ce—C16—O5	101.92 (18)
O1W—Ce—O2—C8	67.98 (17)	O2W—Ce—C16—O5	−77.3 (2)
O2W—Ce—O2—C8	135.25 (18)	O7—Ce—C16—O5	17.85 (16)
O7—Ce—O2—C8	99.2 (2)	O1^ii^—Ce—C16—O5	92.63 (16)
O1^ii^—Ce—O2—C8	1.52 (19)	O2—Ce—C16—O5	−163.42 (15)
O5—Ce—O2—C8	−87.59 (18)	O4—Ce—C16—O5	178.8 (3)
O4—Ce—O2—C8	−70.68 (17)	O8—Ce—C16—O5	−30.32 (16)
O8—Ce—O2—C8	174.30 (16)	O1—Ce—C16—O5	151.73 (15)
O1—Ce—O2—C8	−3.94 (15)	O8^i^—Ce—C16—O4	90.29 (16)
C16—Ce—O2—C8	−78.19 (18)	O1W—Ce—C16—O4	−76.9 (2)
O8^i^—Ce—O4—C16	−83.43 (16)	O2W—Ce—C16—O4	103.89 (19)
O1W—Ce—O4—C16	131.50 (15)	O7—Ce—C16—O4	−160.95 (15)
O2W—Ce—O4—C16	−115.90 (17)	O1^ii^—Ce—C16—O4	−86.18 (16)
O7—Ce—O4—C16	22.76 (18)	O5—Ce—C16—O4	−178.8 (3)
O1^ii^—Ce—O4—C16	86.95 (16)	O2—Ce—C16—O4	17.77 (17)
O5—Ce—O4—C16	0.66 (14)	O8—Ce—C16—O4	150.87 (16)
O2—Ce—O4—C16	−163.09 (16)	O1—Ce—C16—O4	−27.07 (16)
O8—Ce—O4—C16	−31.83 (17)	C22—C17—C18—C19	−0.6 (5)
O1—Ce—O4—C16	150.88 (17)	C23—C17—C18—C19	−178.7 (3)
O8^i^—Ce—O5—C16	82.91 (16)	O9—C12A—C19—C18	−179.7 (3)
O1W—Ce—O5—C16	−115.47 (17)	C21—C12A—C19—C18	1.6 (5)
O2W—Ce—O5—C16	132.19 (16)	C17—C18—C19—C12A	−0.6 (5)
O7—Ce—O5—C16	−161.91 (17)	O9—C12A—C21—C22	179.9 (3)
O1^ii^—Ce—O5—C16	−81.21 (16)	C19—C12A—C21—C22	−1.3 (6)
O2—Ce—O5—C16	21.44 (19)	C18—C17—C22—C21	0.9 (5)
O4—Ce—O5—C16	−0.66 (14)	C23—C17—C22—C21	179.0 (3)
O8—Ce—O5—C16	147.35 (17)	C12A—C21—C22—C17	0.1 (6)
O1—Ce—O5—C16	−31.07 (17)	C22—C17—C23—C24	58.3 (5)
O8^i^—Ce—O7—C24	−2.2 (2)	C18—C17—C23—C24	−123.7 (4)
O1W—Ce—O7—C24	133.2 (2)	Ce—O7—C24—O8	−3.1 (3)
O2W—Ce—O7—C24	67.79 (19)	Ce—O7—C24—C23	174.8 (3)
O1^ii^—Ce—O7—C24	−152.7 (2)	Ce^i^—O8—C24—O7	172.5 (3)
O5—Ce—O7—C24	−72.71 (19)	Ce—O8—C24—O7	2.9 (3)
O2—Ce—O7—C24	102.1 (2)	Ce^i^—O8—C24—C23	−5.4 (6)
O4—Ce—O7—C24	−90.04 (19)	Ce—O8—C24—C23	−175.1 (3)
O8—Ce—O7—C24	1.64 (17)	C17—C23—C24—O7	31.7 (5)
O1—Ce—O7—C24	177.85 (17)	C17—C23—C24—O8	−150.3 (3)
C16—Ce—O7—C24	−80.40 (19)	C29—N1—C25—C26	3.2 (7)
O8^i^—Ce—O8—C24	174.3 (2)	N1—C25—C26—C27	−2.5 (8)
O1W—Ce—O8—C24	−53.15 (18)	C25—C26—C27—C28	0.4 (7)
O2W—Ce—O8—C24	−103.16 (18)	C25—C26—C27—C32	179.5 (4)
O7—Ce—O8—C24	−1.60 (16)	C26—C27—C28—C29	0.6 (6)
O1^ii^—Ce—O8—C24	28.4 (2)	C32—C27—C28—C29	−178.4 (3)
O5—Ce—O8—C24	88.10 (17)	C25—N1—C29—C28	−2.0 (6)
O2—Ce—O8—C24	−143.46 (17)	C27—C28—C29—N1	0.2 (6)
O4—Ce—O8—C24	114.31 (17)	C34—N2—C30—C31	−0.5 (8)
C16—Ce—O8—C24	101.32 (17)	N2—C30—C31—C32	0.9 (8)
O8^i^—Ce—O8—Ce^i^	0.0	C30—C31—C32—C33	−0.9 (7)
O1W—Ce—O8—Ce^i^	132.55 (9)	C30—C31—C32—C27	178.0 (4)
O2W—Ce—O8—Ce^i^	82.54 (10)	C26—C27—C32—C31	−33.7 (6)
O7—Ce—O8—Ce^i^	−175.89 (14)	C28—C27—C32—C31	145.2 (4)
O1^ii^—Ce—O8—Ce^i^	−145.87 (7)	C26—C27—C32—C33	145.2 (4)
O5—Ce—O8—Ce^i^	−86.20 (10)	C28—C27—C32—C33	−35.8 (5)
O2—Ce—O8—Ce^i^	42.25 (13)	C31—C32—C33—C34	0.7 (6)
O4—Ce—O8—Ce^i^	−59.98 (11)	C27—C32—C33—C34	−178.3 (4)
C16—Ce—O8—Ce^i^	−72.97 (10)	C30—N2—C34—C33	0.3 (7)
C6—C1—C2—C3	−0.3 (6)	C32—C33—C34—N2	−0.4 (7)
C7—C1—C2—C3	178.4 (4)	C39—N3—C35—C36	−0.2 (7)
C1—C2—C3—C4	−1.1 (7)	N3—C35—C36—C37	0.4 (7)
C2—C3—C4—O3	−177.8 (4)	C35—C36—C37—C38	0.3 (6)
C2—C3—C4—C5	1.5 (7)	C35—C36—C37—C42	−176.6 (4)
O3—C4—C5—C6	178.8 (4)	C36—C37—C38—C39	−1.0 (6)
C3—C4—C5—C6	−0.5 (7)	C42—C37—C38—C39	175.9 (4)
C2—C1—C6—C5	1.3 (6)	C35—N3—C39—C38	−0.6 (7)
C7—C1—C6—C5	−177.4 (4)	C37—C38—C39—N3	1.3 (8)
C4—C5—C6—C1	−0.9 (7)	C44—N4—C40—C41	−0.9 (7)
C6—C1—C7—C8	−119.9 (3)	N4—C40—C41—C42	2.3 (7)
C2—C1—C7—C8	61.5 (5)	C40—C41—C42—C43	−2.2 (6)
Ce^ii^—O1—C8—O2	−169.4 (2)	C40—C41—C42—C37	176.7 (4)
Ce—O1—C8—O2	−6.8 (3)	C38—C37—C42—C41	−142.8 (4)
Ce^ii^—O1—C8—C7	7.8 (5)	C36—C37—C42—C41	34.0 (5)
Ce—O1—C8—C7	170.5 (2)	C38—C37—C42—C43	36.1 (5)
Ce—O2—C8—O1	7.7 (3)	C36—C37—C42—C43	−147.2 (4)
Ce—O2—C8—C7	−169.6 (2)	C41—C42—C43—C44	1.0 (6)
C1—C7—C8—O1	122.2 (3)	C37—C42—C43—C44	−177.9 (4)
C1—C7—C8—O2	−60.5 (4)	C40—N4—C44—C43	−0.3 (7)
C14—C9—C10—C11	−0.8 (5)	C42—C43—C44—N4	0.3 (7)

Symmetry codes: (i) −*x*+2, −*y*, −*z*+1; (ii) −*x*+1, −*y*, −*z*+1.

### Hydrogen-bond geometry (Å, °)

**Table:**

*D*—H···*A*	*D*—H	H···*A*	*D*···*A*	*D*—H···*A*
O1W—H1···O4^ii^	0.81	1.96	2.765 (3)	172
O1W—H7···N1^ii^	0.83	2.02	2.774 (4)	151
O2W—H2···O5^i^	0.82	1.89	2.703 (3)	177
O2W—H5···O3W^ii^	0.82	2.16	2.924 (3)	155
O3—H15A···N3^iii^	0.86	1.94	2.781 (4)	168
O6—H6A···O3W^iv^	0.82	1.87	2.684 (3)	172
O9—H9A···N2^v^	0.80	1.91	2.700 (5)	168
O3W—H10···O2^ii^	0.85	1.91	2.752 (3)	168
O3W—H11···N4^vi^	0.81	2.01	2.817 (4)	175

Symmetry codes: (ii) −*x*+1, −*y*, −*z*+1; (i) −*x*+2, −*y*, −*z*+1; (iii) −*x*+2, −*y*+1, −*z*; (iv) −*x*+1, −*y*+1, −*z*+1; (v) *x*, *y*−1, *z*+1; (vi) −*x*, −*y*+1, −*z*+1.
